# *In silico* prospecting of the mtDNA of *Macrobrachium amazonicum* from transcriptome data

**DOI:** 10.1186/s12864-023-09770-y

**Published:** 2023-11-10

**Authors:** Jerônimo Costa Marques-Neto, Gabriel Monteiro de Lima, Carlos Murilo Tenório Maciel, Bruna Ramalho Maciel, Fernando Araujo Abrunhosa, Iracilda Sampaio, Cristiana Ramalho Maciel

**Affiliations:** 1 Laboratory of Aquaculture, Coastal Studies Institute, Federal University of Pará, Alameda Leandro Ribeiro S/N, Aldeia, Bragança, Pará CEP: 68600-000 Brazil; 2 Coastal Studies Institute, School of Biological Sciences, Laboratory of Aquaculture/BioDatta, Federal University of Pará, Alameda Leandro Ribeiro S/N, Aldeia, Bragança, Pará CEP: 68600-000 Brazil; 3 Coastal Studies Institute, School of Biological Sciences, Laboratory of Carcinology, Federal University of Pará, Alameda Leandro Ribeiro S/N, Aldeia, Bragança, Pará CEP: 68600-000 Brazil; 4 Coastal Studies Institute, Federal University of Pará, Alameda Leandro Ribeiro S/N, Aldeia, Bragança, Pará CEP: 68600-000 Brazil

**Keywords:** Freshwater prawn, Mitogenome, Coding regions, Molecular analysis

## Abstract

**Background:**

*Macrobrachium amazonicum* is a freshwater prawn widely distributed in South America that is undergoing speciation, so the denomination “*M. amazonicum* complex” is used for it. The mitochondrial cytochrome *c* oxidase subunit I (COI) gene has been used to elucidate this speciation, but heteroplasmies and pseudogenes have been recorded, making separation difficult. Obtaining genes from cDNA (RNA) rather than genomic DNA is an effective tool to mitigate those two types of occurrences. The aim of this study was to assemble in silico the mitochondrial DNA (mtDNA) of the Amazonian coastal population of *M. amazonicum* inhabiting the state of Pará.

**Results:**

Sequences were obtained from the prawn’s transcriptome using the de novo approach. Six libraries of cDNA from the androgen gland, hepatopancreas, and muscle tissue were used. The mtDNA of *M. amazonicum* was 14,960 bp in length. It contained 13 protein-coding genes, 21 complete transfer RNAs, and the 12S and 16S subunits of ribosomal RNA. All regions were found on the light strand except tRNA^Gln^, which was on the heavy strand. The control region (D-loop) was not recovered, making for a gap of 793 bp. The cladogram showed the formation of the well-defined *Macrobrachium* clade, with high support value in the established branches (91–100). The three-dimensional spatial conformation of the mtDNA-encoded proteins showed that most of them were mainly composed of major α-helices that typically shows in those proteins inserted in the membrane (mitochondrial).

**Conclusions:**

It was possible to assemble a large part of the mitochondrial genome of *M. amazonicum *in silico using data from other genomes deposited in GenBank and to validate it through the similarities between its COI and 16S genes and those from animals of the same region deposited in GenBank. Depositing the *M. amazonicum* mtDNA sequences in GenBank may help solve the taxonomic problems recorded for the species, in addition to providing complete sequences of candidate coding genes for use as biomarkers in ecological studies.

## Introduction

The freshwater prawn *Macrobrachium amazonicum* (Heller, 1862) has a wide distribution across South America and adapts well to coastal and continental water bodies, including channels, lakes, rivers, and reservoirs [1–4]. Anger (2013) [5] attributes to the variations between populations a species-complex status – the *M. amazonicum* complex – which is undergoing speciation. Studies along these lines have addressed both morphological aspects and molecular biology, but the separation of this complex has not yet been fully established [6–9].

To date, only the population of the Brazilian Pantanal is considered separate due to molecular evidence, and peculiarities in its ontogenetic development and morphology [7, 9]. It is considered a new species called *M. pantanalense* [7]. Recently, it was found that specimens of *M. amazonicum* collected in the Tietê River (São Paulo state) and *M. pantanalense* collected in the Baiazinha Lagoon (Miranda, Mato Grasso do Sul state—Pantanal) could not copulate, reinforcing the speciation hypothesis for this population [10]. For the others, their speciation status is not yet known, and they continue to be grouped in the *M. amazonicum* complex. In an attempt to better describe the *M. amazonicum* complex, fragments of only two mitochondrial genes have been used: cytochrome *c* oxidase subunit I and the 16S ribosomal RNA gene [6, 9], and there is no record of a complete mitochondrial genome.

Most prawn mitochondrial genomes present in GenBank belong to other genera. Only five complete mitogenomes have been registered for *Macrobrachium*, of which three were published in the literature [11–13]. Obtaining the mitochondrial genome of the Amazon river prawn is one viable way to start answering questions about the taxonomy of the species.

Mitochondrial DNA (mtDNA) is located in the inner membrane matrix and is similar to a bacterial chromosome, being circular and highly compact [14]. It has uniparental inheritance and undergoes changes in its nucleotide composition at a relatively rapid rate [15–17]. It differs in size between eukaryotic species but usually has the same total number of regions, 37, of which 13 contain genes responsible for protein coding (cytochrome oxidase subunit I, II and III; ATP synthase subunits 6 and 8; nicotinamide adenine dinucleotide dehydrogenase (NADH) subunits 1, 2, 3, 4, 5, 6, and 4L; and cytochrome *b*), 22 encode transfer RNAs (tRNAs), and two encode ribosomal RNA subunits (one large,16S, and one small, 12S) [18, 19]. Some of these genes are used as bioindicators of the presence of chemical compounds in the body, whereas others can be used as tools for phylogenetic analysis, arousing interest in obtaining the complete mtDNA genomes from many species [20, 21].

Molecular analyses using mitochondrial genes have helped solve some taxonomic problems, including in prawn species of the genus *Macrobrachium* Spence Bate, 1868, because these species are very diverse, have a wide distribution, and have complex evolutionary histories [5, 22]. There are records of phenotypic variations, causing problems in the separation of species within the genus [23–25].

There are several ways to obtain the complete mtDNA molecule, the most traditional of which is DNA purification, using specific primers, followed by Sanger sequencing [26]. Over the past 10 years, the number of functional genome (transcriptome) libraries of various species deposited in public databases has increased [27, 28]. Recently, the mitochondrial genome of several species was obtained through in silico prospecting in databases of mitochondrial RNA sequenced in transcriptomes, using bioinformatics tools [29, 30]. The use of transcriptome data linked to the de novo method has aroused interest as an assembly tool due to its speed and accuracy when editing mitogenomes [31]. *M. amazonicum* of the Amazonian coast has a sequenced functional genome, allowing the search for its mitochondrial genes in databases. The aim of this study is to assemble the mtDNA genome of this population using the de novo assembly approach in silico.

## Methods

### Sampling

The databases of six cDNA libraries were obtained from androgen gland (AG), hepatopancreas (HEP), and muscle (MUS) tissue and sequenced on the Illumina HiSeq 2500 platform (Illumina, San Diego, CA, USA). Each library was constructed from a pool of 10 animals, with two replicates each. *M. amazonicum* has evident and significant genetic divergence among populations, structuring them in three groups: I- inland waters of the Amazonian Hydrographic Region (HR); II- Paraná/Paraguay HR; and III- coastal systems of northern and northeastern Brazil [6]. The animals used were descendants of the individuals collected from the third population (01°12′37.7′′S, 46°08′17.1′′W).

### mtDNA assembly and data analysis

The *M. amazonicum* transcriptome databases were assembled using Trinity assembler [32] without performing the filtering step that removes ribosomal genes because there are genes of this category in the mtDNA. Filtering was performed to remove low-quality sequences (< 20) using FastQC [33] (which analyses the sequences from the transcriptome) and Trimmomatic 0.39 [34] (which is used for trimming bases and sequences). The assembly was performed by the de novo method without a reference genome. Then, the mtDNA regions were searched for, using as reference the mitochondrial genome of the *M. rosenbergii* congener (NCBI AY659990.1), which is phylogenetically close to the target species.

The MEB program (Maciel, 2015 – not published) was used to search for sequences with higher similarities using the BLASTn tool, in which the six databases of the *M. amazonicum* transcriptome were compared against the 37 mtDNA sequences of *M. rosenbergii.* The results were visualized in Notepad +  + v. 7.8.4 [35].

The sequences identified as possible regions of the mitogenome of *M. amazonicum* were then automatically aligned in BioEdit v. 7.2.5 [36] with the aid of the ClustalW tool [37], taking the complete genome of *M. rosenbergii* as a reference. To determine the positions of the mtDNA regions identified in the Amazon river prawn, in order to construct a consensus and complete mitogenome, all sequences previously identified with the MEB program were used.

The start/stop codon and amino acid composition of the 13 protein-coding genes (PCGs) were identified by the ORFfinder tool. The content of the nitrogenous bases was automatically quantified in BioEdit v. 7.2.5. The closed circular representation of the mtDNA was created using the online tool Genome Vx (http://wolfe.ucd.ie/GenomeVx/) and was edited in Adobe Photoshop CC 2018 (version 19.1.6.).

Two divergence matrices were generated. In the first, ~ 85% of the mitogenome of seven different prawn species was used, in which the cutting position occurred at the beginning of the control region (not recovered in the *M. amazonicum* transcriptome). In the second matrix, the sequences of the protein-coding regions were used to quantify the differences between *M. amazonicum* and five other species of the genus. This analysis was based on the model of Kimura (1980) [38] and was performed with the aid of MEGA X software [39]. The maximum likelihood tree was also generated in MEGA X using the GTR evolutionary model with 1,000 pseudoreplicates, and ~ 85% of the mitochondrial genome of the prawn species was also used. The three-dimensional structures of each PCG were drawn in SWISS-MODEL software [40] from the amino acid sequence using ENDscript 2.0 to identify the α-helices and β-sheets [41] and PyMOL [42] for later editing.

## Results and discussion

### General arrangement of the mitochondrial genome

**Fig. 1:**
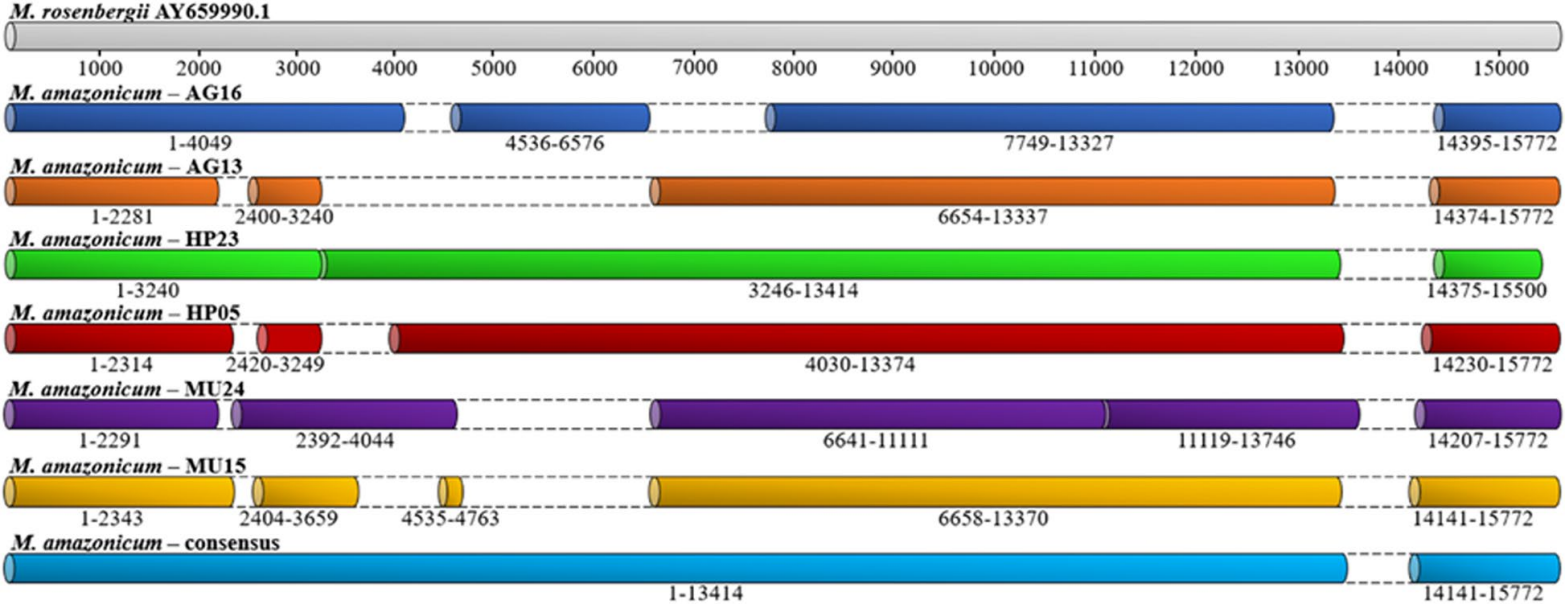
Databases used in the assembly of the mitogenome of *M. amazonicum*. The genome of the reference species is shown (top, in light gray); the androgen gland (AG), hepatopancreas (HP) and muscle (MU) libraries; and the final consensus sequence (light blue) with the recovered transcripts. The bp size is shown. Unrecovered sites are represented by white space between dashed lines in the databases and in the consensus sequence

The use of the six transcriptome databases (in silico), combined with the de novo assembly method, optimized the recovery of more than 90% of the *M. amazonicum* mitogenome (Fig. 1), yielding a 14,960-bp fragment, indicating that this is a satisfactory way to assemble mitochondrial genomes. In addition, this method offers cost savings, agility, and good coverage of regions because it can be performed by mining databases deposited in GenBank or similar databases [43–45].

The total adenine and thymine (A + T) content was higher (61.33%) than that of guanine and cytosine G + C (38.67%) in the mtDNA. The percentages of the four bases in the whole mitogenome and its parts are listed in Table 1. The A + T content was similar to that of the mitogenome of the congener species used for comparison, suggesting it may be a characteristic of this genus [11–13].

**Table 1:** Percentage of nitrogenous bases by regions in the mitochondrial genome of *M. amazonicum*

*M. amazonicum*	Size (bp)	A%	C%	G%	T%	A + T%	G + C%
Mitogenome	14,960	36.21	25.49	13.18	25.12	61.33	38.67
PCGs	11,196	36.02	26.97	12.80	24.21	60.23	39.77
tRNAs	1418	34.56	20.58	14.63	30.24	64.80	35.20
rRNA	2143	39.41	21.39	10.18	29.03	68.44	31.57

A, C, G, and T contents in the whole mitochondrial genome of *M. amazonicum* and in its protein-coding regions (PCGs), transfer RNAs (tRNAs), and ribosomal RNAs (rRNA). The noncoding spaces are accounted for only in the value of the complete genome, and gene overlaps were removed from the final values

**Fig. 2:**
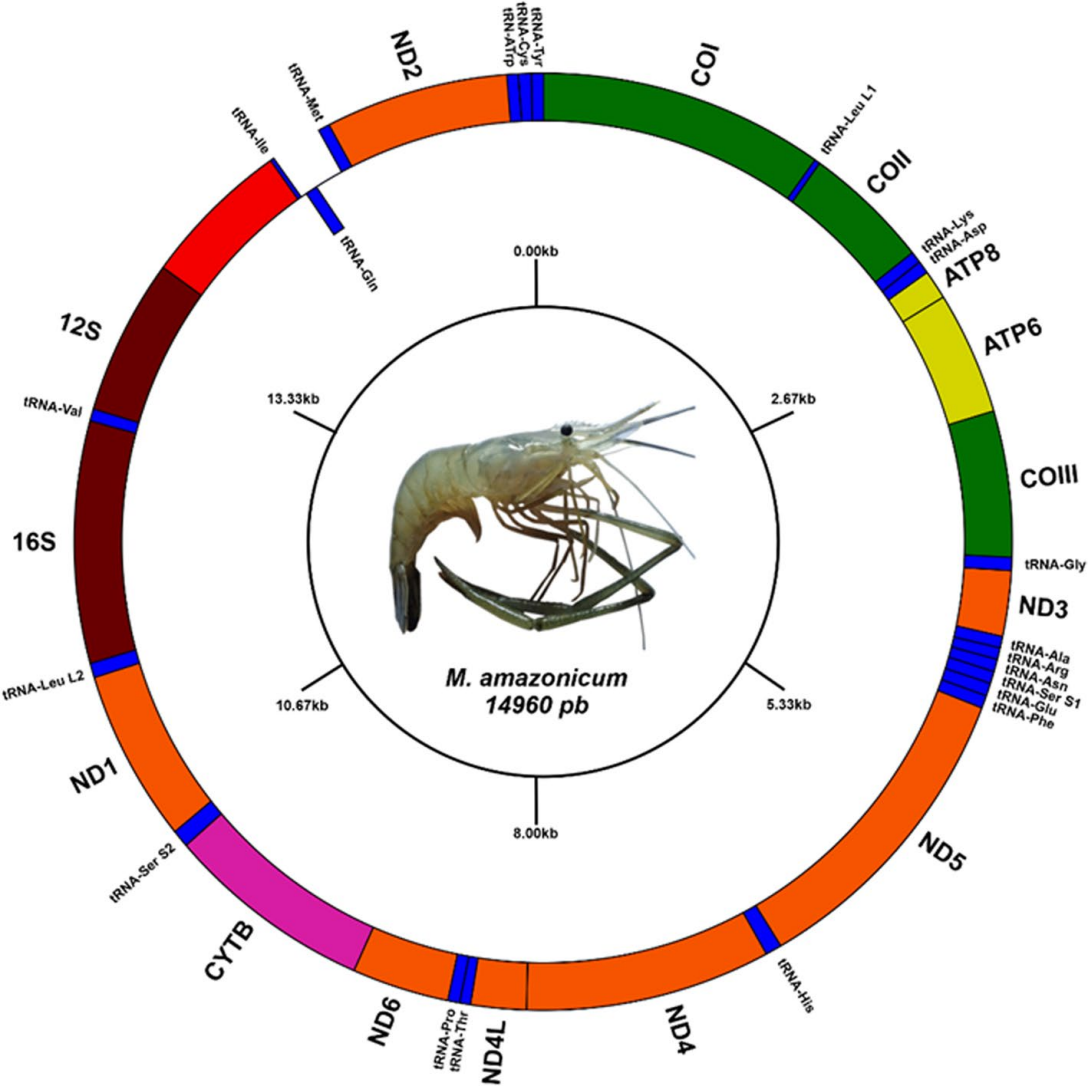
Gene map showing the organization of the mitogenome regions of *M. amazonicum*. The three cytochrome *c* oxidase (COI/II/III) genes are in green, ATP6 and ATP8 in yellow, the six NADH subunits (1, 2, 3, 4, 4L, 5, 6) in orange, cytochrome *b* in pink, the two ribosomal RNA subunits in brown (16S and 12S), and the transfer RNAs in blue. The latter are differentiated by the three-letter codes corresponding to the amino acid name. The heavy strand is denoted by the position of tRNA^Gln^. The light strand has most of the genes. The noncoding sites are spaced and not filled. The middle circle indicates the kilobase (kb) values, and the inner circle indicates the total number of bases recovered from the mtDNA of *M. amazonicum*

All 13 regions encoding common proteins in eukaryotes were found, as were 21 complete tRNAs and two rRNA subunits (12S and 16S) (Fig. 2). The noncoding control region (D-loop) was not recorded in any of the six transcriptomes analysed, and nor were the 41 bases at the beginning of the tRNA^Ile^ that followed the site of the D-loop, together making up an unrecovered 793-bp portion (Fig. 1). Moreira et al. (2015) [46] previously reported the absence of the D-loop in an assembly made from transcriptomic data in a fish species of the family Loricariidae. According to those authors, this was due to the low number of supporting reads of the region in the transcriptomic data, which was directly related to the role of the D-loop in replication/transcription, culminating in its absence from mature transcripts. Therefore, our results do not deviate from the pattern identified in the literature; however, this region can be recovered using specific primers in short-range PCR and subsequently sequenced by the Sanger method [47].

### Region locations and characteristics

The light strand encoded all regions except tRNA^Gly^, which was located in the heavy strand (H) (Fig. 1 and Table 2). As described by Anderson et al. (1981) [48], this condition is related to the amounts of G and T in each of the strands; the more of these two bases there are, the heavier it will be. This difference in the percentage of bases between the strands is attributed to the replication process because this step occurs asynchronously, which facilitates the emergence of mutations when nucleotides are added or removed at the end of the process [49, 50]. In the present study, the percentage of bases revealed a prevalence of A and C in the mtDNA of *M. amazonicum*, explaining the presence of most regions in the light strand.

**Table 2:** Gene components of mitochondrial DNA of *M. amazonicum*

Gene	Position	Size (bp)	Codon (aa)		Strand (L = light, H = heavy)	aa^1^	In^2^
			**Start (aa)**	**Stop**			
COI	1–1560	1560	ACG (T)	TAG	L	519	0
tRNA^Leu (L1)^	1561–1590	30			L		0
COI	1591–2286	696	CTT (L)	TAA	L	231	0
tRNA^Lys^	2285–2354	68			L		0
tRNA^Asp^	2355–2415	61			L		0
ATP8	2416–2583	168	TAT (Y)	TAA	L	55	-7
ATP6	2577–3251	675	ATG (M)	TAA	L	224	4
COIII	3256–4044	789	ATG (M)	TAA	L	262	0
tRNA^Gly^	4045–4115	71			L		0
ND3	4116–4481	366	ATG (M)	TAG	L	121	-10
tRNA^Ala^	4472–4534	63			L		-1
tRNA^Arg^	4534–4599	66			L		1
tRNA^Asn^	4601–4665	65			L		0
tRNA^Ser (S1)^	4666–4732	67			L		2
tRNA^Glu^	4733–4801	69			L		-1
tRNA^Phe^	4801–4865	65			L		0
ND5	4866–6572	1707	ATG (M)	TAA	L	568	0
tRNA^His^	6573–6655	83			L		0
ND4	6656–7983	1335	ATG (M)	TAA	L	452	-7
ND4L	7984–8283	300	ATG (M)	TAA	L	99	0
tRNA^Thr^	8284–8338	55			L		-1
tRNA^Pro^	8338–8403	66			L		0
ND6	8404–8931	528	ACT (T)	TAA	L	175	-1
Cyt*b*	8931–10067	1137	ATG (M)	TGA	L	378	0
tRNA^Ser (S2)^	10068–10158	85			L		2
ND1	10153–11091	939	ATG (M)	TAG	L	312	0
tRNA^Leu (L2)^	11092–11169	78			L		0
16S	11170–12466	1297			L		0
tRNA^Val^	12467–12,532	66			L		0
12S	12533–13378	846			L		- -
D-loop	- -	- -			L		- -
tRNA^Ile*^	13403–13424	22			L		-29
tRNA^Gln^	13452–13518	67			H		181
tRNA^Met^	13698–13767	70			L		0
ND2	13768–14763	996	ATT(I)	TAA	L	331	-2
tRNA^Trp^	14762–14830	69			L		1
tRNA^Cys^	14832–14894	63			L		0
tRNA^Tyr^	14895–14960	66			L		0

Listed are the position; size (bp); codons, in which the amino acid triplet is followed by a letter in parentheses indicating the first amino acid in each sequence: M (methionine), T (threonine), L (leucine), D (aspartic acid), I (isoleucine), Y (tyrosine); strand (light or heavy); number of amino acids (aa^1^) and intergenic nucleotides (In^2^), where positive values indicate noncoding spaces in the mitogenome and negative values indicate the overlap of two regions. The asterisk (*) indicates the incomplete tRNA

Regarding the intergenic regions, seven gene overlaps were found between regions, the largest 29 nt (tRNA^Ile^ – tRNA ^Gln^) and the smallest 1 nt (Table 2). Small noncoding spaces were observed throughout the mitogenome, the most extensive being between the tRNA^Gln^ and tRNA^Met^ regions (181 nt) and the smallest located between tRNA^Ile^ and tRNA^Gln^ (28 nt) (Table 2). Both overlaps and noncoding spaces are characteristics observed in the mtDNA of many crustaceans [51–53] but are not exclusive to the phylum. The lack of synchrony of the strands during the replication process, generating nucleotide sequences that do not encode any transcript, is well documented in other groups [54–56]. In addition, the small size of these fragments indicates that they do not play an important role in the mtDNA, unlike the control region, which has the key role of holding the necessary promoters for initiating transcription/replication [57].

The total length of all PCGs was 11,196 bp, and the A + T content of this region was 60% (Table 1). The longest gene was ND5: 1707 bp, located between tRNA^Phe^ and tRNA^His^; while the shortest was ATP8: 168 bp, located between tRNA^Asp^ and ATP6. The other 11 genes were ~ 300 to 1560 bp (Table 2). This pattern has also been observed in the mitogenomes of the five species of the genus *Macrobrachium*, in which the largest and smallest regions were also ND5 and ATP8, respectively. The lengths of these two genes were also similar between these species, as were the sites that flanked these genes; this was not observed in other shrimp genera, corroborating the existence of a pattern particular to the genus *Macrobrachium* [11, 12, 58].

The space occupied by the tRNA genes was 1418 bp. The content of the A + T bases was higher (65%) than that of G + C (35%) in these regions (Table 1). The size of most tRNA genes ranged from ~ 60 to 78 bp, and the smallest was 30 bp, that of tRNA^Leu (L1)^ (Tables 1 and 2). When comparing this tRNA with that of the reference species, we observed that the sizes were similar; however, when searching for the amino acid composition of the region preceding this site (COI), we found that part of what was considered Leu L1 included the amino acid triplet equivalent to the stop codon of COI, so its total number of nucleotides was lower than that of *M. rosenbergii*.

The two ribosomal RNA subunit genes had a size of 2143 bp, the 16S gene being longer than the 12S (1297 and 846 bp, respectively). These were separated by tRNA^Val^. Both were located on the light strand and had 68% A + T content (Tables 1 and 2). These findings were similar to those in other *Macrobrachium* mitogenomes, reinforcing the idea of a pattern in the distribution of the genes thus far observed for the genus. These similarities suggest the absence of the gene rearrangement phenomenon for *Macrobrachium*, so the regions are aligned at the same position between species, suggesting that there are no cases of mispairing between the strands [59]. To date, there are only five congeneric species with fully sequenced mtDNA genomes, including the one here. When these data are expanded, we can determine whether these traits are actually conserved throughout the genus.

### Start and stop codons

The start codon ATG, encoding the amino acid methionine, was present at the beginning of eight genes, and the start of transcription is attributed to this triplet, as it is usually considered the start codon of the transcription process, both in vertebrates and in invertebrates [55, 60–62]. Alternative start codons (threonine, leucine, tyrosine, and isoleucine) were recorded in the other genes (Table 2), threonine starting two of the genes: COI and ND6 (ACx codons). Records of these alternative start codons are frequent in the mitochondrial genomes of malacostracans, including the *Macrobrachium* sequences used for comparison in the present study [11, 12, 58, 63–68]. In addition, the consistency of these data between *M. amazonicum* and the six replicate libraries used in the assembly of the mtDNA was verified, showing that the start codons were not random phenomena due to possible inaccuracies in the in silico assembly. Kearse and Wilusz (2017) [69] noted the relevance of this type of event, since translation can be initiated by amino acids other than methionine, which is essential for the regulation of essential processes of protein synthesis.

All 13 PCGs showed functional stop codons (TAG and TAA) (Table 2). This indicates that the release factor (eRF1), which triggers a series of processes that punctuate the end of translation [70], acts on all genes of the *M. amazonicum* mitogenome.

### Divergence matrices and phylogenetic analysis

In the divergence matrix constructed with ~ 85% of the total mitochondrial genome, a divergence pattern within the genus *Macrobrachium* was observed, defined as 17–22%, with the lowest divergence values between species occurring in *M. rosenbergii vs. M. lanchesteri* (17%) and *M. nipponense vs. M. bullatum* (18%). *M. amazonicum* showed the highest divergence levels of all the species of the group, with 21% divergence from *M. rosenbergii*, *M. nipponense*, and *M. bullatum* and 22% divergence from *M. lanchesteri* (Table 3). These values were close to those found in the divergence matrix constructed with the coding genes alone, showing that the genetic distance between *M. amazonicum* and the other five congeners fits within the parameters considered to distinguish species within this genus [23, 24, 71]. Of the coding genes, ND6 (30%) showed the greatest divergence, and ND4L and COI the lowest (18% to 19%) (Table 4).

**Table 3:** Divergence matrix between species of *Macrobrachium* and *Caridian* prawn

	1	2	3	4	5	6	7	8
*M. amazonicum*								
*M. rosenbergii* AY659990.1	0.21							
*M. rosenbergii* KY865098.1	0.21	0.08						
*M. nipponense* HQ830201.1	0.21	0.21	0.21					
*M. lanchesteri* FJ797435.1	0.22	0.17	0.17	0.20				
*M. bullatum* KM978918.1	0.21	0.21	0.21	0.18	0.21			
*E. carinicauda* EF560650.1	0.35	0.36	0.36	0.35	0.35	0.35		
*R. variabilis* MH714460.1	0.36	0.37	0.37	0.35	0.36	0.36	0.39	

Mitogenome of *M. amazonicum* with five other congeners + two species of Caridean prawn, using ~ 85% of the mitochondrial genome of each species (cut after the control region, which was not recovered for *M. amazonicum*)

**Table 4:** Divergence matrix of the 13 mitochondrial protein-coding genes identified in *M. amazonicum*

	***M. amazonicum***												
	**COI**	**COII**	**ATP8**	**ATP6**	**COIII**	**ND3**	**ND5**	**ND4**	**ND4L**	**ND6**	**Cyt*b***	**ND1**	**ND2**
*M. rosenbergii* AY659990.1	0.19	0.18	0.26	0.22	0.22	0.33	0.26	0.26	0.19	0.33	0.21	0.19	0.29
*M. rosenbergii* KY865098.1	0.19	0.20	0.28	0.20	0.21	0.26	0.26	0.25	0.18	0.31	0.21	0.20	0.29
*M. nipponense* HQ830201.1	0.18	0.20	0.26	0.26	0.19	0.27	0.27	0.24	0.19	0.32	0.17	0.21	0.32
*M. lanchesteri* KM978918.1	0.21	0.21	0.37	0.27	0.22	0.30	0.26	0.23	0.19	0.34	0.22	0.22	0.32
*M. bullatum* FJ797435.1	0.17	0.20	0.37	0.22	0.19	0.27	0.27	0.25	0.20	0.37	0.21	0.19	0.31

The genes of *M. amazoncium* were compared with the same genes of the five species of the genus that have a complete mitochondrial genome deposited in GenBank. The regions are in the published mitogenomes of the listed species. The adopted values range from 0 to 1: the closer to 1, the greater the divergence

Population studies carried out among the three populations/clades identified for freshwater prawn, using partial regions of the mitochondrial COI gene, showed nucleotide divergences of around 0–3.3% and 0–0.1% for the COI and 16S rRNA genes, while divergences recorded at the interspecific level were 4.8–14.7% and 13.3–19.9% [6], respectively. Previous studies carried out in the *Macrobrachium* complex suggest a minimum divergence above 10% of the COI gene to distinguish species at the molecular level, identifying variations of 0–3.2% and 0–12.6% at the population level, for 16S rRNA and COI [23, 24], corroborating the findings for the three groupings of *M. amazonicum* in the literature [6].

Work carried out between different populations of the genus using partial regions of the mitochondrial COI gene show divergence percentages of 0 to ~ 10% [23, 24, 71], like those recorded for *M. rosenbergii* [72–75], populations of *M. nipponense* [76], *M. australiense* [77] and *M. amazonicum* [6, 78]. However, there is a complex of cryptic species recorded for the genus, necessitating caution when drawing conclusions [24, 79–82].

Another difficulty for phylogenetic studies and species separation in *Macrobrachium *is the existence of synonymies, heteroplasmies, and pseudogenes, which are relatively frequent [24, 83–85]. Numts are copies of the mtDNA in the nuclear DNA [86], and mitochondrial heteroplasmy are more than one mitochondrial genome in the same organism [87]. Numts not only make sequence analysis difficult, but sometimes the sequences may be erroneously adopted as genuine mtDNA sequences. Iketani et al. (2021) [85] frequently detected these events in individuals of the *M. amazonicum* complex, demonstrating the need to use more than one gene for analyses between different populations and the need for caution in drawing conclusions based exclusively on the divergences recorded for the COI gene. To circumvent the effect of Numts, the use of mtDNA-rich tissue, mtDNA enrichment, dilution of template DNA, protein-coding regions, long PCR, or cDNA analysis has been recommended [85, 88]. Kang et al. (2016) [89], when using cDNA (RNA) sequences from the orthopteran species *Anapodisma miramae*, they found that heteroplasmies and pseudogenes, common in the species, had practically disappeared. In the present study, all mtDNA genes found originated from RNA purifications, increasing the reliability of the results.

When the regions of the COI and 16S rRNA genes recorded in the mitogenome assembled in the present study were compared with the sequences generated by Vergamini et al. (2011) [6], we found ~ 99 to 100% similarity with the haplotypes corresponding to the population of Abaetetuba, Pará state (GU929471.1 and GU929450.1, respectively). These data show the accuracy of the in silico assembly of the mtDNA of *M. amazonicum* because the animals used for transcriptome sequencing came from offspring from nearby locations, in communicating water bodies. In this sense, the recovery of the 13 PCGs from the mitogenome of the coastal population of *M. amazonicum* (including COI, 16S rRNA, and 12S rRNA) may enable the use of alternative mtDNA genes in future population studies of the species, such as Cytb, ATP6, ATP8, COII, COIII, and ND2. These genes show conservation of their nucleotide content, in addition to consistent variation between different species [90–93], which can assist in the resolution of the incongruence and taxonomic problems of the *M. amazonicum* complex.

**Fig. 3:**
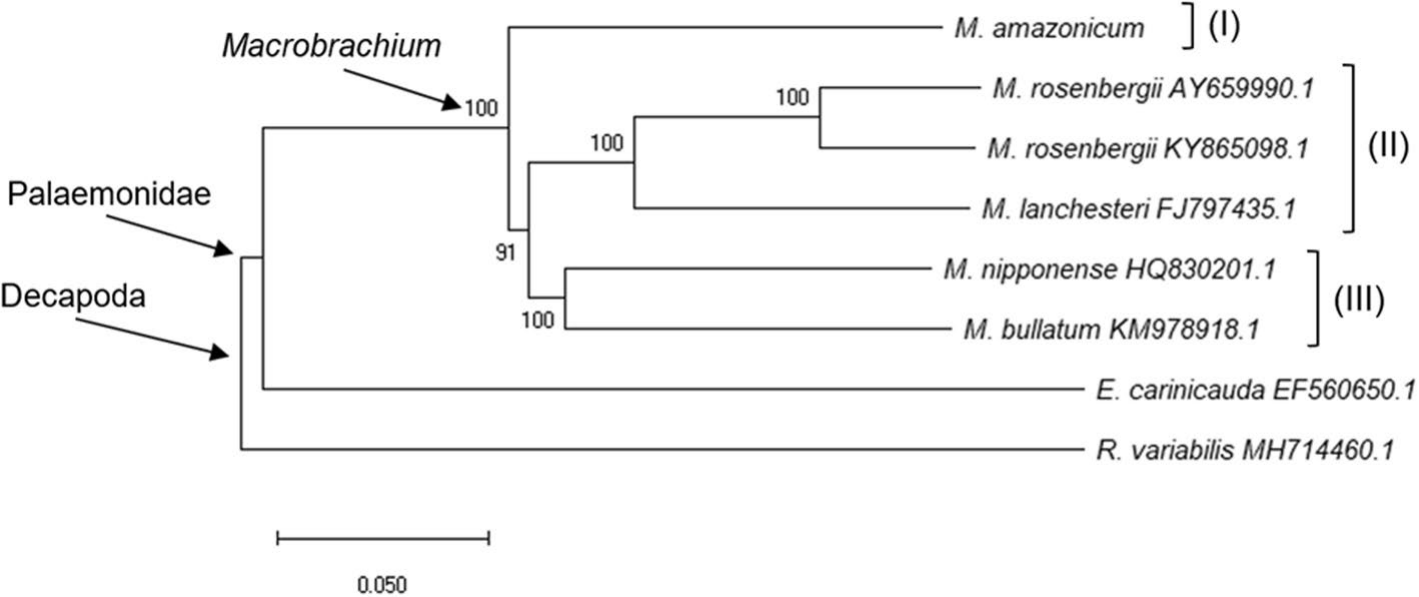
Cladogram showing the relationships of the group Decapoda based on mitogenomic data with 1,000 pseudo replicates. The numbers in the branches indicate the bootstrap values. The GenBank accession code is next to each species

The cladogram showed the formation of the well-defined *Macrobrachium* clade, with high support value in the established inner branches (91–100), using maximum likelihood with a bootstrapping of 1,000 pseudoreplicates. *Macrobrachium* showed the formation of three groups, *M. amazonicum* (I), *M. rosenbergii* + *M. lanchesteri* (II), and *M. nipponense* + *M. bullatum* (III). The species *Exopalaemon carinicauda* formed a group external to the analysed genus, with 35–36% divergence from *Macrobrachium*, while *Rimicaris variabilis* was located external to the family Palaemonidae, showing 35–39% divergence from the other taxa (Fig. 3 and Table 3).

Due to the lack of standardization of the precise divergence percentages required for species separation, other aspects end up being considered, such as morphological and biological data. For the Amazon river prawn, three main populations have been established in Brazil (Amazon, North–Northeast coast, and Paraná–Paraguay watersheds), with maximum intraspecific divergences of 3.3% and 0.1% for the COI and 16S rRNA genes, respectively [6]. However, the Pantanal population (Paraná–Paraguay watershed), which presents ~ 3% divergence, was given a new species assignation, *Macrobrachium pantanalense* [7], taking into account mainly the variations in morphological, reproductive, and physiological aspects [8, 10, 94–97].

### Three-dimensional protein structures

Most proteins had three-dimensional conformations rich in α-helices. Berg et al. (2014) [98] reported that the percentage of this type of structure can vary considerably in the final arrangement of a secondary composition. The three-dimensional spatial conformation of the mtDNA-encoded proteins showed that most of them were mainly composed of major α-helices. They show hydrophobic residues typical in those proteins inserted in the membrane (mitochondrial). In our study, some proteins were composed exclusively of this type of arrangement (COI, ATP6, ATP8, COIII, ND4, ND4 L, ND6, ND1, and ND2), suggesting the existence of internal hydrogen bonds between the carboxyl (CO) end of an amino acid and the amine end (NH) of the other, both located in the main chain, which leads to a recognizable torsional pattern along its axis [99].

Some proteins also had β-sheets (COII, ND3, ND5, and Cytb). This arrangement is common and results from branches in the β-carbon atom of some amino acid, in which case α-helices would not be viable, as this would lead to steric collisions between amino acids with β-branching and NH-CO groups [100].

**Fig. 4:**
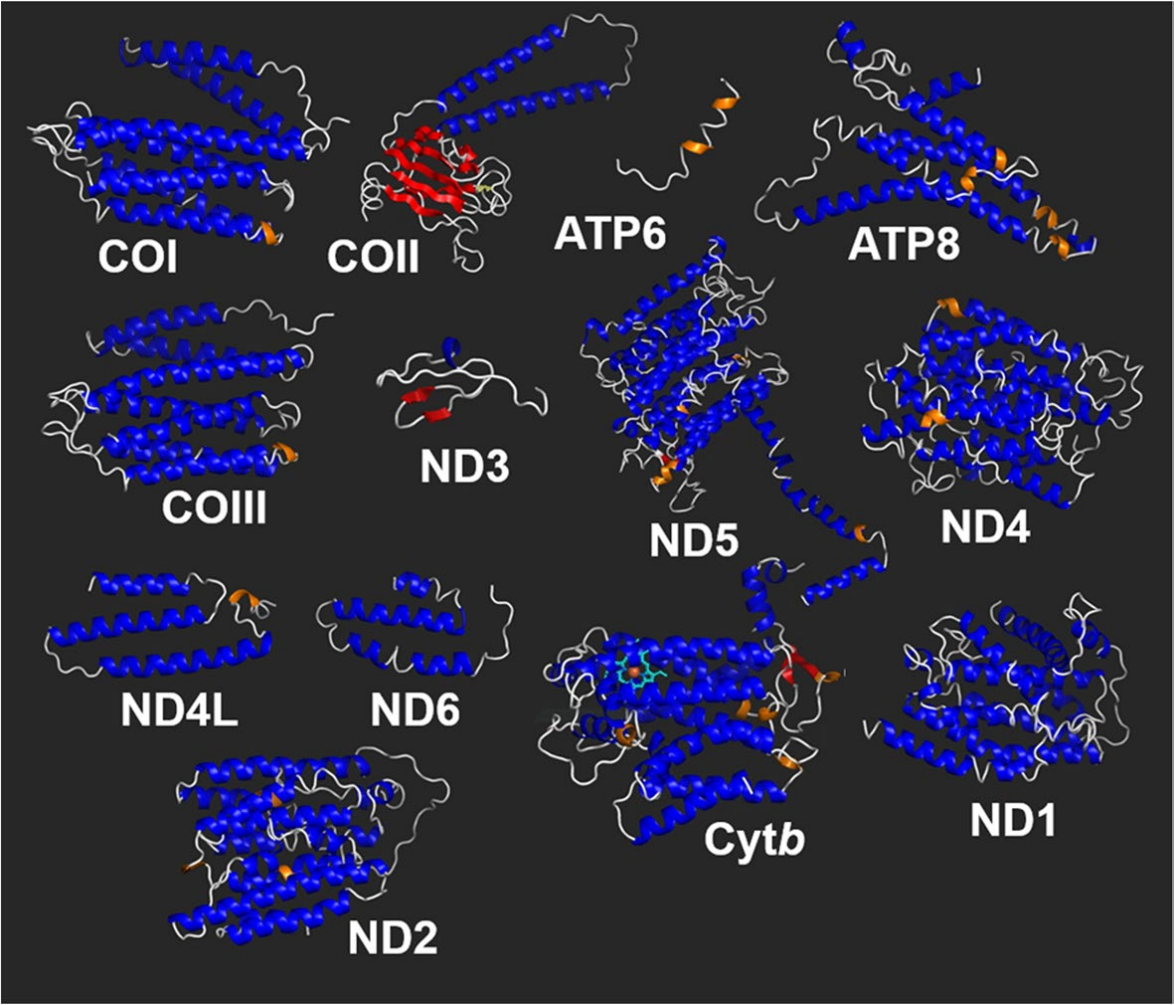
Representations of the three-dimensional structures of the mitochondrial-encoded proteins. A prominent characteristic of the α-helices (blue and orange) is the folding of the polypeptide skeleton around the main chain, while the β-sheets (red) are characterized by hydrogen bonds between the polypeptide chains, presenting a flat and rigid pattern

The most notable particularity was detected in Cyt*b*, corresponding to the presence of the haem group (light blue), attached to an iron atom (orange circle) (Fig. 4). This is characteristic of Cyt*b* and acts in the transfer of the electron of ubiquinone (coenzyme Q) to cytochrome *c* [101, 102], a vital process for cellular activity. The toxic agent cyanide has affinity to the iron binding site, where it interrupts energy production in the cell [103–105]. In addition, the response of mitochondria to stress and different types of pollutants [20, 106–108] make mtDNA a possible bioindicator of environmental quality [21]. Thus, the *M. amazonicum* mitogenome made available in the present study can be used not only for future genetic analyses but also for investigations of ecosystem data, aided by the fact that the species is widely distributed across South America [5].

## Conclusions

The de novo assembly method using transcriptomic data yielded a satisfactory in silico assembly because it was possible to determine the composition, location, and distribution of the mitogenomic regions of the Amazon river prawn. The *M. amazonicum* mtDNA has features similar to that of similar species, but with significant interspecific divergences to distinguish them. The light strand encodes most regions, characterized by its high A + C content, which was one of the starkest differences from the other species of the genus, which have six or eight regions in the heavy strand. The complete recovery of proteins paves the way for using the mitochondrial genome of *M. amazonicum* as an indicator of environmental quality. The database deposited in GenBank, corresponding to the coastal population of the species, will aid in more refined studies on the distinct populations and speciation of the *M. amazonicum* complex.

## Data Availability

The datasets generated and/or analysed during the current study are available in the GenBank repository, https://www.ncbi.nlm.nih.gov/nuccore/ON513382.1, under the accession number ON513382.1.

## Abbreviations

mtDNA: Mitochondrial DNA
tRNAs: RNA carriers
L: Light strand
H: Heavy strand
Ile: Isoleucine
Gln: Glutamine
Met: Methionine
Ala: Alanine
Leu L1: Leucine L1
Leu L2: Leucine L2
His: Histidine
Ser S1: Serine S1
Ser S2: Serine S2
ND2: NADH Dehydrogenase Subunit 2
ND3: NADH 3: NADH Dehydrogenase Subunit 3
ND4: NADH Dehydrogenase Subunit 4
ND4L: NADH Dehydrogenase Subunit 4L
ND5: NADH Dehydrogenase Subunit 5
ND6: NADH Dehydrogenase Subunit 6
COI: Cytochrome Oxidase C Subunit I
GCPs: Protein Encoding Genes
bp: Base pairs
CR: Control Region
